# Supplementary material for: Development and Characterization of Folic Acid-Conjugated Amodiaquine-Loaded Nanoparticles–Efficacy in Cancer Treatment
Source: Pharmaceutics. 2023 Mar 20;15(3):1001. doi: 10.3390/pharmaceutics15031001 (PMC10053199; doi:10.3390/pharmaceutics15031001)
Supplement: Supplementary file 1 [file pharmaceutics-15-01001-s001.zip › pharmaceutics-2208153-supplementary.pdf]

SUPPLEMENTARY INFORMATION

Development and Characterization of Folic Acid-Conjugated Amodiaquine-Loaded Nanoparticles--Efficacy in Cancer Treatment

Vineela Parvathaneni, Snehal K Shukla, and Vivek Gupta

Methods

Development of UPLC method for Determination of Amodiaquine (AQ)

A reverse-phase liquid chromatography method was developed for quantifying AQ using Waters series Acquity UPLC (Waters, USA). The column used was Xselect™ HSS T3 (3.0 × 100 mm; 2.5 µm particles). The mobile phase consisted of aqueous phase of 0.1% orthophosphoric acid in water, and organic phase of ACN at 50:50 at a flow rate of 0.5 ml/min at 343 nm wavelength. Retention time was found to be 0.733 min with total run time of 1.5 min. Data were collected and analyzed using the Empower 3.0 software.

Formulation of Non-Targeted (AQ NP Rota) and FA-Targeted AQ NPs (FA-AQ NPs):

Briefly, coarse emulsion was prepared using probe homogenization and then processed through Nano DeBee® HPH (BEE International, South Easton, MA, USA) The obtained emulsion was subjected for overnight stirring to remove organic solvent. Next day, nanoparticles were washed twice using 1× PBS by centrifugation at 21,000 RCF for 15 min, to remove excess PVA and unprocessed drug/polymer. Due to less solubility of AQ in PBS, it was optimized as the dilution medium for PVA to reduce drug leaching during washing process. Collected nanoparticles were re-dispersed in milliQ water (0.5mL). To elaborate, after completion of HPH processing, collected formulation (45mL) have been subjected for overnight stirring and then collected nanoparticle

pellet has been redispersed in 0.5mL of milliQ water to obtain liquid nanoparticle formulation. Later, formulations were subjected for rotary vacuum evaporation for organic solvent removal followed by washing of nanoparticles for removal of excess PVA. Then, nanoparticles were washed for removal of excess PVA and were redispersed in milliQ water.

Morphological Analysis:

Imaging of unconjugated and conjugated AQ nanoparticles was performed using transmission electron microscopy (TEM) for assessing nanoparticles' morphology. Formvar® carbon-coated copper grids 400-mesh (Electron Microscopy Sciences, PA, USA) were made hydrophilic using glow discharge plasma treatment for 60s. About 5 µL of diluted nanoparticles sample was added to the grid and allowed to adhere for 1 minute. The samples were negatively stained by adding 5 µL of uranyl acetate (1%) and excess was removed by using Whatman filter paper after 30s. This process was repeated twice, and the sample was finally allowed to air dry. The grids were then imaged using FEI Tecnai Spirit TWIN TEM (FEI, Hillsboro, OR, USA) operated at 120 kV voltage.

Solid-state Characterization Studies

Powder X-ray Diffraction (PXRD) Studies: The samples were first lyophilized into dry powders using Labconco FreeZone® freeze dryer system. X-ray diffraction spectroscopy was carried out using XRD-6000 (Shimadzu, Kyoto, Japan). diffractometry was performed by using a graphite monochromator consisting of copper-Kα1 radiation of wavelength 1.5418 Å operating at 40 kV, 30 mA. The samples were spread uniformly on a glass micro sample holder and were analyzed in the range of 5 to 60° at the scanning speed of 2° (2θ)/minute.

Differential Scanning Calorimetry (DSC) Studies: The thermograms for AQ, AQ NP Rota and FA-AQ NP were generated by closed pan technique using a DSC 6000 (PerkinElmer, Inc; CT, USA) equipped with an intra-cooler accessory. An accurately weighed sample (2mg) was sealed in an aluminum pan and analyzed against a sealed empty aluminum pan maintained as reference. Study was performed over a temperature range of 30°- 300°C The heating rate was maintained at 10°C/min.

Cellular Uptake (Microscopic Assessment):

In brief, A549 cells were plated in 8-chambered tissue culture (TC) treated cell imaging cover glass (Eppendorf, Hauppauge, NY, USA) at a seeding density of 10,000 cells/chamber followed by overnight incubation. Next day, cells were incubated with coumarin-6, coumarin-6 loaded unconjugated and FA conjugated nanoparticles at 1 μg/mL concentration in fresh culture medium for 3 hours. After 3 hours, cells were washed with ice cold PBS twice and fixed with 4% paraformaldehyde (PFA) for 10 min. Fixed cells were washed again with ice cold PBS twice. Then, the chamber was removed and 20μL of vectashield hardset mount with DAPI nuclear stain (H1500, Vector laboratories, Burlingame, CA, UAS) was placed on a glass slide dropwise followed by placing a cover glass. After hardening of mounting medium overnight at 4°C, slides were imaged using EVOS-FL microscope at 20X magnification (Thermo Scientific, Waltham, MA, USA).

Cellular Uptake (Quantitative Assessment by Determining Fluorescence Intensity):

Briefly, cells were seeded into 24-well assay plates at 1×10^5 cells/well. Next day, cells were incubated them with 1 ml at 1μg/ml equivalent concentration of coumarin, coumarin-loaded NPs and FA- conjugated coumarin nanoparticles at 5% CO_2/37°C for 3 hours. After 3 hours, media was removed from the wells and washed three times with ice-cold PBS. Cells were harvested using trypsin and to the obtained cell pellets, 100 μl of 0.5% Triton X-100 was added for cell lysis. These

samples were kept for sonication for one hour at 4°C. The fluorescence intensity of supernatants from each sample was measured for coumarin quantification at Ex/Em of 420/465nm (Tecan Spark 10M; Tecan, Männedorf, Switzerland).

Cytotoxicity Studies:

AQ NP Rota, FA-AQ NPs along with plain AQ were evaluated for their cytotoxicity in multiple cancer cell lines: A549 (non-small cell lung cancer, NSCLC); HeLa (cervical cancer) and MDAMB-231 (breast cancer) as reported earlier with slight modifications [1,2]. HeLa and MDAMB-231 cell lines were chosen because of their feature to exhibit overexpression of folic acid receptors [3]. In brief, A549 cells were grown in FBS supplemented RPMI-1640 media and HeLa, MDAMB-231 cells were grown in FBS supplemented DMEM media as described in *Materials* section, and were seeded in TC treated 96-well plates (Eppendorf, Hauppauge, NY, USA) at a seeding density of 2,500 cells/well (7,500 cells/cm^2), incubated overnight for adherence at 37°C/5% CO_2, and treatments were added next day at different AQ concentrations ranging from 0.39-50 μM. Corresponding volumes of AQ NP Rota and FA-AQ NPs were calculated based on the drug entrapment efficiency. Blank culture media was added as control. After 72 hours of incubation, % cell viability was determined by performing MTT assay as described earlier [4], by reading the absorbance of dissolved formazan crystals at 570 nm (Tecan Spark 10M; Tecan, Männedorf, Switzerland). Further cytotoxicity studies were performed following same protocol to evaluate the safety of drug-free nanoparticles (HEK) using equivalent amounts of Blank NP at concentrations of 0.39, 0.78, 1.56, 3.13, 6.25, 12.5, 25 and 50μM with 72 hours of incubation period.

Clonogenic Assay:

Clonogenic assay is an *in-vitro* cell survival assay which is based on the single cell's capability to grow into a colony. A colony forming assay was performed to evaluate long-term cytotoxicity of AQ, AQ NP Rota and FA-AQ NP toward colony inhibition was determined. The protocol reported previously [2,5] was briefly modified and followed in this study. HeLa or MDAMB-231 cells were seeded in 6-well cell culture plates at seeding density of 500 cells/well for each cell line. After overnight incubation and adherence of cells, cells were subjected to AQ, AQ NP Rota and FA-AQ NP (10µM), or control treatments for 48 hours after which media was replaced with fresh culture medium on alternative days for 7 days. On 7th day, formed colonies were washed with ice-cold PBS and stained with 0.01% (w/v) crystal violet followed by washing with distilled water as reported earlier. Colonies were photographed using digital camera and the mean number of colonies from three independent experiments were counted by colony counter software Open CFU [6].

Live-Dead Cell Assay:

Briefly, 100µL of 2µM calcein AM/4µM Ethidium homodimer III (EthD-III) staining solution was added to spheroids after complete removal of media from the respective wells. The plate was incubated for 45 min at room temperature. This provides green/red fluorescent staining of viable and dead cells, respectively. Images were captured using (EVOS-FL, Thermo Fisher Scientific) and mean red fluorescence intensity/mm^2 of spheroid was quantified using ImageJ software which signifies the presence of live cells in spheroid mass.

Results and Discussion

Synthesis of PLGA-PEG-FA:

PLGA-PEG-FA was synthesized by following the previously reported protocol [7]. Details are provided in *Supplementary Information*. Synthesis of PLGA-PEG-FA conjugate was carried out via a set of reactions to activate PLGA, followed by subsequent conjugation reactions presents more reactive γ-COOH group of FA which facilitates binding of FA with the folate receptor as reported earlier by Valencia *et al* [8]. The chemistry presented here for the synthesis of PLGA-PEG-FA allowed for the conjugation of targeting ligand, FA. In brief, activated PLGA was conjugated with PEG moiety which is further conjugated with carboxyl group of FA via its amine group. **Fig. 1B** outlines the synthesis procedure of PLGA-PEG-FA.

References

[1] N.S. Kulkarni, V. Parvathaneni, S.K. Shukla, L. Barasa, J.C. Perron, S. Yoganathan, A. Muth, V. Gupta, Tyrosine kinase inhibitor conjugated quantum dots for non-small cell lung cancer (NSCLC) treatment, Eur. J. Pharm. Sci. Off. J. Eur. Fed. Pharm. Sci. 133 (2019) 145–159. https://doi.org/10.1016/j.ejps.2019.03.026.

[2] B. Vaidya, V. Parvathaneni, N.S. Kulkarni, S.K. Shukla, J.K. Damon, A. Sarode, D. Kanabar, J.V. Garcia, S. Mitragotri, A. Muth, V. Gupta, Cyclodextrin modified erlotinib loaded PLGA nanoparticles for improved therapeutic efficacy against non-small cell lung cancer, Int. J. Biol. Macromol. 122 (2019) 338–347. https://doi.org/10.1016/j.ijbiomac.2018.10.181.

[3] Y. Yu, J. Wang, S.C. Kaul, R. Wadhwa, E. Miyako, Folic Acid Receptor-Mediated Targeting Enhances the Cytotoxicity, Efficacy, and Selectivity of Withania somnifera Leaf Extract: In vitro and in vivo Evidence, Front. Oncol. 9 (2019). https://doi.org/10.3389/fonc.2019.00602.

[4] V. Parvathaneni, N.S. Kulkarni, S.K. Shukla, P.T. Farrales, N.K. Kunda, A. Muth, V. Gupta, Systematic Development and Optimization of Inhalable Pirfenidone Liposomes for Non-Small Cell Lung Cancer Treatment, Pharmaceutics. 12 (2020). https://doi.org/10.3390/pharmaceutics12030206.

[5] N.A.P. Franken, H.M. Rodermond, J. Stap, J. Haveman, C. van Bree, Clonogenic assay of cells in vitro, Nat. Protoc. 1 (2006) 2315–2319. https://doi.org/10.1038/nprot.2006.339.

[6] Q. Geissmann, OpenCFU, a New Free and Open-Source Software to Count Cell Colonies and Other Circular Objects, PLOS ONE. 8 (2013) e54072. https://doi.org/10.1371/journal.pone.0054072.

[7] F. Esmaeili, M.H. Ghahremani, S.N. Ostad, F. Atyabi, M. Seyedabadi, M.R. Malekshahi, M. Amini, R. Dinarvand, Folate-receptor-targeted delivery of docetaxel nanoparticles prepared by PLGA–PEG–folate conjugate, J. Drug Target. 16 (2008) 415–423. https://doi.org/10.1080/10611860802088630.

[8] P.M. Valencia, M.H. Hanewich-Hollatz, W. Gao, F. Karim, R. Langer, R. Karnik, O.C. Farokhzad, Effects of ligands with different water solubilities on self-assembly and properties of targeted nanoparticles, Biomaterials. 32 (2011) 6226–6233. https://doi.org/10.1016/j.biomaterials.2011.04.078.
